# Early Congenital Cytomegalovirus Detection Pathways in Pediatric Audiology Services in England: Findings From a National Audit in England

**DOI:** 10.1097/INF.0000000000004496

**Published:** 2024-09-11

**Authors:** Vishnuga Raveendran, Hannah Garnett, Catherine Magee, Simone Walter, Heather Bailey

**Affiliations:** * Institute for Global Health, University College London, London, United Kingdom; † National Health Service (NHS) England, London, United Kingdom; ‡ St George’s University Hospitals National Health Service (NHS) Foundation Trust, London, United Kingdom

## To the Editors:

Sensorineural hearing loss (SNHL) is present at birth or occurs later in childhood in ≥40% of children born with symptoms of congenital cytomegalovirus (cCMV) and up to 30% of cCMV-infected infants who are “asymptomatic” at birth.^1^ Trial data have shown modest benefits of antiviral treatment to hearing and development when initiated in symptomatic babies at <4 weeks of age.^2^ Data on treatment benefits in asymptomatic children with isolated SNHL are limited, while possible side effects make shared decision-making important; in this group, recent findings indicate possible benefits to hearing if started at ≤3 months.^3^

In England, newborns are not routinely screened for cCMV, but cCMV-related hearing loss at birth may be identified via newborn hearing screening. Early cCMV detection pathways among infants referred for audiological assessment aim to expedite cCMV diagnosis, to allow prompt decisions around treatment, follow-up and rehabilitation.^4^ Quality Standards in Pediatric Audiology specify that services must have clearly defined pathways for early cCMV diagnosis^5^; however, national availability is unknown. Using data from a national audit, we describe the availability and characteristics of early cCMV detection pathways.

A web-based survey was completed by pediatric audiology departments (1 response per department) and members of the British Association of Audiovestibular Physicians and British Association of Paediatricians in Audiology in England (1 response per clinician investigating the etiology of childhood hearing loss) between November 25, 2022, and January 6, 2023. As this was an audit, ethics approval was not required. Fisher's exact tests were used for categorical comparisons.

Fifty-five pediatric audiology departments responded, and 31 clinicians investigating etiology, representing approximately 42% and 41% of the total in England, respectively. Half [55% (30/55)] of the audiology departments reported access to an early cCMV detection pathway and 68% (21/31) of clinicians investigating etiology (58% of 76 departments with a department and/or clinician response). This proportion was 74% (17/23) of departments with ≥10 permanent childhood hearing impairment diagnoses per year versus 48% (13/27) of smaller departments (*P* = 0.09).

Within early detection pathways, departments reported that cCMV diagnostic samples (most often saliva, 62%) were mostly taken at detection of SNHL in audiology (40%) or by the newborn hearing screener at point of referral (27%) (Table 1). Over a quarter of clinicians reported use of dried blood spots (indicating testing at >3 weeks of age), all of whom indicated access to saliva testing also (Table 1). cCMV test results were available within 48 hours to 60% of audiology departments versus 22% of clinicians. Overall, 65% (22/34) of departments reported having seen ≥1 child with SNHL diagnosed with cCMV <4 weeks of age over the last 5 years and a similar proportion [71% (20/28)] having seen ≥1 child with SNHL diagnosed with cCMV between 4 weeks and 3 months of age.

**TABLE 1.** Number and Proportion [n (%)] of Audiology Departments and Clinicians Reporting Access to an Early cCMV Detection Pathway and, Among Those With a Pathway, Its Characteristics

	Audiology Department Responses	Clinician Responses
Access to an early cCMV detection pathway		
No	20/55 (36%)	7/31 (25%)
Yes	30/55 (55%)	21/31 (68%)
Don’t know	5/55 (9%)	3/31 (14%)
Stage of early cCMV detection pathway CMV test sample is taken*		
By the newborn hearing screener at point of referral	8/28 (27%)	6/21 (28%)
By audiologist for all babies referred from newborn hearing screening	1/28 (3%)	2/21 (10%)
At detection of SNHL in audiology	11/28 (40%)	11/21 (52%)
For any baby referred to audiology where hearing not proved normal	5/28 (13%)	1/21 (5%)
When baby is seen for etiologic investigations	3/28 (7%)	1/21 (5%)
cCMV diagnostic samples used*†		
Urine	11/29 (38%)	3/21 (14%)
Saliva	18/29 (62%)	20/21 (95%)
Dried blood spot	1/29 (3%)‡	6/21 (29%)‡
Time taken for CMV test results to be available*		
Within 24 h	1/22 (5%)	2/19 (11%)
25–48 h	12/22 (55%)	2/19 (11%)
2–7 d	8/22 (36%)	14/19 (74%)
>1 wk	1/22 (5%)	1/19 (5%)

* The total number of responses after excluding those without an early cCMV detection pathway and those who answered “don’t know.”

† Multiple responses possible.

‡ All departments and clinicians who reported the use of dried blood spots also reported the use of saliva samples.

CMV indicates cytomegalovirus.

Almost half of the pediatric audiology departments did not have access to an early cCMV detection pathway. The range of pathway designs reflects variations in organization of services and testing availability. This may have implications for inequalities in time frames for cCMV diagnosis and treatment among babies with hearing loss.

## ACKNOWLEDGMENTS

*The authors thank all the respondents to this pediatric audiology audit.*
